# Phage-Bacterial Dynamics with Spatial Structure: Self Organization around Phage Sinks Can Promote Increased Cell Densities

**DOI:** 10.3390/antibiotics7010008

**Published:** 2018-01-29

**Authors:** James J. Bull, Kelly A. Christensen, Carly Scott, Benjamin R. Jack, Cameron J. Crandall, Stephen M. Krone

**Affiliations:** 1 Department of Integrative Biology, University of Texas, Austin, TX 78712, USA; 2 The Institute for Cellular and Molecular Biology, University of Texas, Austin, TX 78712, USA; 3 Center for Computational Biology and Bioinformatics, University of Texas, Austin, TX 78712, USA; 4 Department of Mathematics, University of Idaho, Moscow, ID 83844, USA; chri4898@vandals.uidaho.edu (K.A.C.); scot9278@vandals.uidaho.edu (C.S.); 5 Center for Modeling Complex Interactions, University of Idaho, Moscow, ID 83844, USA; 6 Department of Biological Sciences, University of Idaho, Moscow, ID 83844, USA; cjcrandall91@gmail.com; 7 The Institute for Cellular and Molecular Biology, University of Texas, Austin, TX 78712, USA; benjamin.r.jack@gmail.com; 8 Institute for Bioinformatics and Evolutionary Studies, University of Idaho, Moscow, ID 83844, USA

**Keywords:** biofilm, phage therapy, resistance, bacteriophage, models, agent based, mass action

## Abstract

Bacteria growing on surfaces appear to be profoundly more resistant to control by lytic bacteriophages than do the same cells grown in liquid. Here, we use simulation models to investigate whether spatial structure per se can account for this increased cell density in the presence of phages. A measure is derived for comparing cell densities between growth in spatially structured environments versus well mixed environments (known as mass action). Maintenance of sensitive cells requires some form of phage death; we invoke death mechanisms that are spatially fixed, as if produced by cells. Spatially structured phage death provides cells with a means of protection that can boost cell densities an order of magnitude above that attained under mass action, although the effect is sometimes in the opposite direction. Phage and bacteria self organize into separate refuges, and spatial structure operates so that the phage progeny from a single burst do not have independent fates (as they do with mass action). Phage incur a high loss when invading protected areas that have high cell densities, resulting in greater protection for the cells. By the same metric, mass action dynamics either show no sustained bacterial elevation or oscillate between states of low and high cell densities and an elevated average. The elevated cell densities observed in models with spatial structure do not approach the empirically observed increased density of cells in structured environments with phages (which can be many orders of magnitude), so the empirical phenomenon likely requires additional mechanisms than those analyzed here.

## 1. Introduction

Bacteriophages are ubiquitous predators of bacteria, and they have long been entertained as having possible therapeutic utility in medicine. However, therapeutic utility is typically a matter of controlling the bacterial populations, and population control is not easily inferred from the mere fact that individuals of one species can kill individuals of another species. The difference between killing that achieves population control and killing that has little effect on the population rests on quantitative properties of the killing. Fortunately, phages are easily manipulated in the lab and thus easily studied to address dynamics and the control of bacterial populations.

The history of work on phage-bacterial dynamics has been dominated by liquid cultures in which bacteria are suspended as single cells at uniform density. Such cultures are routinely modeled as ordinary differential equations (ODEs) with assumptions of “mass action.” Mass action refers to an environment in which all individuals are “well mixed,” as would occur in a chemostat or batch culture, and so collisions occur at random. In such a system (see Equation (7)), interaction terms appear as products of bulk densities and essential parameters are easily estimated. The typical outcome following a lytic phage assault on a dense population of sensitive bacteria in liquid is killing of the bacterial population by many orders of magnitude, followed by a rebound of bacteria genetically resistant to the phage [1,2] with possible long-term coevolutionary arms races [3]. This work has led to many insights about bacterial and bacteriophage biology but has also given rise to a perception that bacterial escape from phages is chiefly through evolution of genetic resistance. However, we now know that many bacteria spend much of their lives in structured environments such as biofilms and aggregates, and bacterial biology in structured environments is fundamentally different than in liquid suspensions [4,5,6]. Spatially structured bacterial populations are difficult to control—they may persist seemingly indefinitely amid ongoing phage attack (they also survive antibiotic attack), and this persistence does not appear to be from genetic resistance [7,8,9,10,11,12,13]. Understanding the nature of this coexistence may be critical to phage therapy. Is it spatial structure itself that allows bacterial escape, or is it an indirect consequence of spatial structure on bacterial habits that allows the escape?

The goal of this study is to use models to understand the maintenance of high densities of sensitive bacteria amid phage attack in spatially structured environments. Our ultimate motivation is to develop phage interventions for controlling bacteria, which requires understanding of how bacteria normally escape. Does spatial structure per se allow for easy persistence, or does escape require cells to behave differently in structured environments than in liquid ones? We use computational models to explore the dynamic nature of the phage-bacterial interaction in spatially structured populations, identifying which mechanisms enable bacterial persistence at high densities. The empirical evidence is that sensitive bacteria easily persist, but identifying a process that may reasonably account for the coexistence is challenging.

## 2. Empirical Anomalies and Possible Causes

Various observations on bacteria grown under spatial structure suggest that genetically sensitive bacteria can be maintained as the dominant population in the presence of phage, at least in the short term [8,10,11,12,14]. The environmental contexts in these examples are diverse. The phage typically reduce bacterial numbers 1 or more orders of magnitude, but the remaining population is predominantly sensitive and persists at a much higher density than would occur in liquid. The phage sensitivity of residual populations is sometimes measured directly or is inferred from dynamic principles, such as the continuing high output of phage (which could not grow on genetically resistant cells). In some cases, the surviving bacterial strain is a genetic mutant that is fundamentally sensitive to phage but exhibits reduced adsorption (e.g., mucoidy); the bacteria are merely maintained at higher levels than explicable by basic dynamics principles (e.g., [15]).

As one striking example, Darch et al. [14] grew *Pseudomonas aeruginosa* in a synthetic sputum medium; cell numbers were measured non-destructively with confocal microscopy. The cells grew in aggregates. Addition of phage to an established culture resulted in a less than 1-log drop in bacterial numbers (measured in situ). However, when the bacteria were grown in liquid (albeit in different media), addition of phage resulted in a 7-log drop. In a second example, Lu and Colins [10] grew 24 h *E. coli* biofilms in peg-lid microtiter plates (0.2 mL volumes per well). After media replacement, 24 h treatment with phage T7 led to approximately a 2-log reduction in cell density, but close to $10^{5}$ cells remained (their Fig. 3B). However, treatment with a T7 phage engineered to encode an enzyme that degrades a bacterial matrix component led to another nearly 2-log reduction in cell density. Density of the enzyme-free phage was $\approx 5\times 10^{8}$/mL in the surrounding liquid. The fact that the enzyme had such a profound effect indicates that sensitive cells were sequestered from the no-enzyme phage while surrounded with a phage density that should have been more than sufficient to eliminate nearly all of them.

Compared to mass action, the most obvious consequence of spatial structure is local variation in the abundance of bacteria and phage. However, this spatial variation arises, reproduction of phage and bacteria enhances that variation, whereas diffusion diminishes it. Structure leads to expanding concentrations of bacteria (colonies) and to high concentrations of phages near bacterial clusters that have been invaded [16,17,18]. The spatial variation in abundance will interact with any of several factors that could be contributors to the long-term co-maintenance of sensitive bacteria and lytic phages, as follows.

**Resource concentration**. Phage growth is known to be reduced on cells that are starved [19,20], a phenomenon easily appreciated from the halting of plaque growth on plates after the bacterial lawn matures. In spatial environments, high concentrations of bacteria will depress resources locally, suppressing phage growth in those zones.
**Barriers and gradients**. Spatial structure allows the local buildup of substances exuded from cells, such as expolysaccharides (EPS), ions, signalling molecules, and outer membrane vesicles [1,8,21]. These agents may trap phages, drive phages away with electrostatic forces, or alter the concentration of factors necessary for phage adsorption.
**Phage-adsorbing debris**. The remnants of cells lysed by phages may continue to adsorb phage perhaps irreversibly and thereby reduce the number of phage encountering live cells. Spatial structure will facilitate the buildup of debris around clusters of cells.
**Co-infection and superinfection**. Phage growth with spatial structure will often concentrate phages around cells, which for many phages will lead to high numbers of phages infecting the same cell [18]. This property will reduce the effective number of phage progeny and may allow cells to reach higher densities than in liquid.
**Altered gene expression**. Cells may vary gene expression specifically in response to surface attachment or signals received from adjacent cells (e.g., [22]). Changes in gene expression are not necessarily effects of spatially structured dynamics per se, but gene expression changes may themselves enable phage-bacterial co-existence. As an example, non-genetic variation in receptor abundance on cells can lead to high levels of the survival of genetically sensitive bacteria challenged with phages [23,24,25,26]. If bacterial growth with spatial structure amplifies variation in gene expression, that variation could enable bacterial escape and subsequent growth, more than in liquid.

## 3. Perspective: Does Spatial Structure Increase Bacterial Density?

The question addressed here is whether phage and cell dynamics that are spatial in nature allow cells to attain a higher density than if everything is well mixed. As our approach uses mathematical and computational models, this question requires understanding the difference between spatial structure and well-mixed conditions. Phage dynamics have traditionally been modeled under the assumptions of mass action, which assumes cells and phages are fully mixed and that interactions occur at rates determined by population averages. Mass action means that cells and phage have no assigned locations; they just exist. This mathematical convenience allows the process to be studied with ordinary differential equations [1,27,28,29]. With spatial structure, the locations of cells and phages are tracked over time, and interactions are location dependent. Typically, phages move through diffusion and cells remain in fixed locations (adjacent to parent cells). Thus, high densities of cells or phages can build up in parts of the environment while other parts have few or no individuals. Phage killing is local to the areas of high phage density.

Extensive computational analyses of spatially structured phage-bacterial dynamics have been undertaken in a few previous studies [16,17,18]. This pioneering work described many properties of dynamics unique to spatial structure, such as strong spatial co-localization of bacteria and phage, as well as spatial structure enabling coexistence over a wider range of parameter values than does mass action (due to greater oscillations with mass action).

Our study uses that foundation to ask a specific question: does spatially structured phage dynamics per se maintain a greater cell density than under mass action? The fact that spatial structure more easily allows coexistence [17] might suggest that spatial structure also increases bacterial density, but the effect of spatial structure was reported to stem from reduced global oscillations rather than an increase in (mean) bacterial density. Reduced oscillations could lead to greater coexistence without affecting mean density.

The reason for using models to study these processes is to develop understanding that cannot feasibly be obtained from empirical studies alone. The models allow control of variables so that effects of single variables can be isolated. From there, one may proceed to empirical studies to test specific processes.

## 4. Setting the Stage for Evaluating the Effect of Spatial Structure: Biological Consequences of Mass Action Are Well Studied

We use a variety of computational approaches to understand phage-bacterial dynamics in spatially structured environments. Whereas the outcomes of simulations are easy to interpret, understanding the causal parameters can be challenging because of the many environmental details that must simultaneously be specified to model dynamics in space. To help understand simulation results, and especially to motivate the types of analyses done with simulations, we offer a brief review of specific mass action results from previous studies using ordinary differential equations.

1. Mass action does not preclude high cell density. Although the typical pattern of phage-bacterial dynamics under mass action is one in which phages decimate the bacterial populations, there are mass action conditions in which high densities of sensitive bacteria can be maintained, typically with a low adsorption rate [28].
2. Maintenance of phages and bacteria requires some form of phage death. The ODE models typically assume a constant rate of phage death or clearance from the system.
3. Numerical solutions to the equations often exhibit undamped and even accelerating oscillations [17,28,29]. The oscillations complicate comparisons of cell density across systems (see below).

## 5. Formal Spatial Structure

We use computational simulations to consider the formal dynamics of phage and bacteria with spatial structure. Our simulations were based on a two-dimensional ‘grid’ of sites and included a mix of stochastic (random) and deterministic processes. In these models, every cell, phage or other agent has a location on the grid; at each time step, infection, reproduction and movement may occur (explained in Methods). These models have many components similar to those in mass action models, but with explicit spatial structure and rates that are locally determined. We are primarily interested in whether and how spatial structure affects the cell density maintained in the presence of phages. The grid models include versions that enforce spatial structure as well as mass action versions, although nearly all trials assumed spatial structure. In the mass action versions of the grid models, each individual gets relocated every time step.

Biologically, there are two general types of bacterial avoidance of phages that may be entertained. One is that bacteria are protected from phages, whether by reduced adsorption rate or by surrounding themselves with anti-phage protection. The second is that cells either produce or associate with phage-killing products but are otherwise intrinsically susceptible when phages encounter them. We focus on the latter here, chiefly because it is non-trivial. It is otherwise clear that fully protected bacteria will be able to grow to the limits permitted by the environment—as is well known from ODE models allowing evolution of genetically resistant bacteria. If spatially structured cell growth combined with phage death does not intrinsically promote higher bacterial densities by several orders of magnitude, then protection of individual bacteria becomes plausible as the main driver.

A challenge in switching from mass action to spatial structure lies in accommodating attachment of phage to bacteria. With mass action models, an adsorption rate coefficient (*k*) subsumes both the chance encounter of a bacteria with phage and the rate at which the phage sticks to the bacterium given an encounter [1]. With spatial structure, we are forced to separate encounter from attachment because the two processes are operating at different scales in different parts of the environment [16].

Although some types of physiological protection of cells may be imposed by the environment (e.g., temperature, metal ions that affect adsorption, pH), of interest here is how the bacteria can potentially influence the local environment to enact protection by blocking encounter with phages. Excretion of extracellular polysaccharides and other substances may directly slow or block phage access altogether, and some of the extracellular matrix may effectively kill phages by binding them irreversibly. Dead cells and outer membrane vesicles may act as decoys that bind phages and cause them to eject their genomes.

## 6. Results

The maintenance of sensitive cells amid phage attack depends fundamentally on phage density and thus on phage death mechanisms. In a closed environment with cells and phages, such as a flask, the absence of phage death (or other form of permanent loss/sequestration) will ensure that phage ultimately eliminate all sensitive cells. Once cells are abundant, even phages with poor adsorption rates will ultimately increase to such densities that cells are rapidly eliminated. In the absence of cells being completely protected from phage, some form of phage death is required to prevent the ultimate buildup of phage to the point that all cells are killed. While it is obvious that fully protected cells can grow with impunity in the presence of phages, it is less obvious how the interplay between phage growth and death will collaborate to allow coexistence of sensitive cells and phage. The latter is our focus here—how phage death mechanisms influence the density of cells maintained.

### 6.1. The Nature of Phage Death Used Here: EPS and Cellular Debris

We will model two phage death mechanisms: adsorption to exopolysaccharides (EPS) and adsorption to dead cells (debris). The main difference in implementation of these two mechanisms is that EPS is treated as a spatially static and permanent mechanism of phage death; debris is also assumed to be spatially static, but its creation waxes and wanes as phage kill more or fewer cells, and it is not permanent, instead having an intrinsic decay rate. The association of debris with phage abundance may lead to substantially different outcomes than with a static phage sink. EPS will be the mechanism employed in all but the last set of studies presented here (for reasons explained below).

We accept that the empirical evidence from liquid cultures does not support a major role of debris in causing phage death (e.g., phage titers in lysed cultures are often stable for months—even when the lysate is not filtered or cleared of bacterial debris—J.J. Bull personal observations). The implementation of death by debris is offered in the spirit of any mechanism that rises and falls with phage attack on cells. Furthermore, if debris is short-lived, it may have an impact but the mechanism be difficult to detect empirically. We note that our mechanisms of phage death do not necessarily obey any empirically established process, mostly for lack of effort to detect such processes. Nonetheless, our assumed processes are seemingly more realistic than the usual assumption of a constant, intrinsic phage death rate, and they fall within the broad realm of mechanisms that cells can use to potentially kill off phages (e.g., outer membrane vesicles). It will be shown that our assumption of a fixed level of permanent EPS is equivalent to a constant phage death rate in mass action models.

Spatial structure will alter the dynamics in several ways [16,17,18], and indeed, it is likely that different models of spatial structure will do so differently. Most fundamentally, a lack of uniform densities will often result, allowing cells to amplify in zones that are temporarily phage-free. As regards phage death, phage reproduction from individual cells will have progeny phage spatially clustered at least temporarily and thus subject to a common fate. In addition, cells may find refuge and amplify behind materials that bind phage and act as phage sinks.

### 6.2. A Formal Measure of Whether Cell Density Is Elevated

If spatial structure leads to an elevated cell density above that with complete mixing (mass action), it might seem sufficient to merely observe cell density alone. However, any comparison of cell densities between spatial structure and mass action is not straightforward, in part because there is no single cell density expected under mass action—the cell density, even at equilibrium, depends on many parameters, such as phage burst size, adsorption rate, and death rate, to mention a few. Complicating matters further, mass action processes can themselves lead to a high cell density at equilibrium. Thus, cell density alone cannot tell us whether spatial structure elevates cell density. The effect of spatial structure must be measured via some comparison to cell density in the absence of spatial structure, a comparison that otherwise avoids confounding the many differences between the two types of models.

One such approach is to directly compare cell density when spatial structure is present to that when it is absent in the simulation; abolishing spatial structure can be done by increasing the diffusion rates of phage and cells [16,17]. This approach is free of alternative interpretations, but it has the drawback that bacterial and phage numbers often oscillate with mass action [16,17,28]. Given the limited dynamical range of cell densities afforded by the simulations, bacteria may often go extinct in the simulations even when the equilibrium density is well above extinction (see below).

We adopt a related approach, one that takes advantage of a universal property of equilibrium under mass action, at which phage and bacterial densities are unchanging. Our approach identifies a reproduction number constant that will be used to scale bacterial densities, with a similar use in [29]. Every successful phage infection of a cell will, on average, lead to one new successful infection. This dynamical property of populations in reproductive equilibrium is commonly used in ecology [30]. In the context of phage-bacterial dynamics under mass action, it means that the following equality holds:

(1) $$\begin{array}{c} \frac{\mathrm{rate}\;\mathrm{of}\;\mathrm{productive}\;\mathrm{phage}\;\mathrm{infection}}{\mathrm{all}\;\mathrm{sources}\;\mathrm{of}\;\mathrm{phage}\;\mathrm{loss}\;\mathrm{from}\;\mathrm{the}\;\mathrm{free}\;\mathrm{state}}\times \;\mathrm{phage}\;\mathrm{fecundity}\;\mathrm{per}\;\mathrm{infection}\;=\;1. \end{array}$$

The ratio on the LHS (left hand side) is merely the fraction of all rates leading to phage loss that result in phage reproduction. Since only one phage offspring from an infected cell will go to establish a new successful infection, the product equals unity on average. We denote the ratio on the LHS of Equation (1) as α. Phage fecundity per infection, known as burst size, is represented here as *b*. We have analytically confirmed that αb=1 at equilibrium in various mass action models (e.g., those in [27,28,31]) and not found any that violate the equality.

For the specific sources of phage loss in the spatial models, we propose

(2) $$\alpha =\frac{k_{C}C}{k_{C}C+k_{I}I+k_{D}D+k_{E}E}$$

where C,I,D,E represent the densities of uninfected cells, infected cells, debris, and EPS, and *k* with appropriate subscripts denotes the various attachment/infection probabilities. The time-variable quantities in Equation (2) are C,I, and *D*, but not all models here allow infection of *I* and *D*; moreover, α is an increasing function of *C* and a decreasing function of *I* and *D*.

In this implementation, α is calculated with the parameters used and values observed in the simulations of spatial structure, but the value of α is otherwise interpreted as that which would obtain if the population obeyed mass action. In particular, the quantities in Equation (2) are calculated globally, ignoring the spatial structure that played a role in their generation. The extent to which αb exceeds 1 then measures the effect of spatial structure in conspiring to allow a higher density of cells than would accrue without spatial structure. It indicates, in effect, the added degree of protection experienced by cells in a spatial setting. If, for example, the current value is αb=5 in a spatial simulation, this should be interpreted to mean that if the system suddenly transformed to mass action dynamics, the phage progeny from a burst would infect an average of five uninfected cells. (Of course, this excess of infections would be sustained only briefly.) We have qualitatively confirmed this behavior with spatial simulations that had equilibrated by suddenly (in the middle of the simulation run) increasing phage diffusion and allowing cells to move as an approximation to mass action. Finally, in any trial, the maximum possible value of αb is *b*, but arbitrarily large values of *b* can be tested for compatibility with cell maintenance.

The observed αb in spatially structured trials is not a measure of cell density directly. However, in the absence of debris attachment ($k_{D}=0$) and superinfection of infected cells ($k_{I}=0$), it may be used to calculate the equilibrium cell density expected under mass action. From Equation (2), the cell density satisfying αb=1 is

(3) $$\begin{array}{c} \hat{C}=\frac{k_{E}E}{k_{C}\left(b-1\right)}. \end{array}$$

$\hat{C}$ provides a constant baseline against which the observed cell density ($C_{o}$) may be compared under the above assumptions. The amplification of cell density due to spatial structure (what we will denote as $A_{g}$, for the grid model, in anticipation of defining an *A* for a second model) is thus the ratio of observed cell density to $\hat{C}$:

(4) $$A_{g}=\frac{C_{o}}{\hat{C}}.$$

$A_{g}$ is dimensionless, thus does not depend on cell density units. For convenience, and to emancipate the results from specific values of grid size, cell densities will be measured as the fraction of patches in the grid occupied by cells.

It is evident from inspection of (4) that $A_{g}$ must have an upper bound ($A_{ub,g}$) whenever cell density has an upper bound. In our model, the upper bound does not arise from grid size, rather it stems from the maximum ratio of cells to EPS:

(5) $$A_{ub,g}=\frac{1}{\hat{C}}\;=\frac{k_{C}\left(b-1\right)}{k_{E}E},$$

where *E* is measured as the fraction of the grid occupied by EPS and the 1 in $1/\hat{C}$ is for a grid filled with cells.

The foregoing applies only if the causes of phage death are unchanging. When superinfection occurs or debris traps phages,

(6) $$\hat{C}\;=\;\frac{k_{I}I+k_{D}D+k_{E}E}{k_{C}\left(b-1\right)}.$$

As *I* and *D* are dynamic variables, their values will not generally be the same at the mass action equilibrium as at equilibrium with spatial structure. The calculation of $\hat{C}$ when superinfection and/or debris are admitted, and thus requires some means of determining those values; it may be possible to put bounds on them, however.

### 6.3. Simulations

#### 6.3.1. Increased Cell Densities Especially with Large Burst Sizes

**Figure 1:**
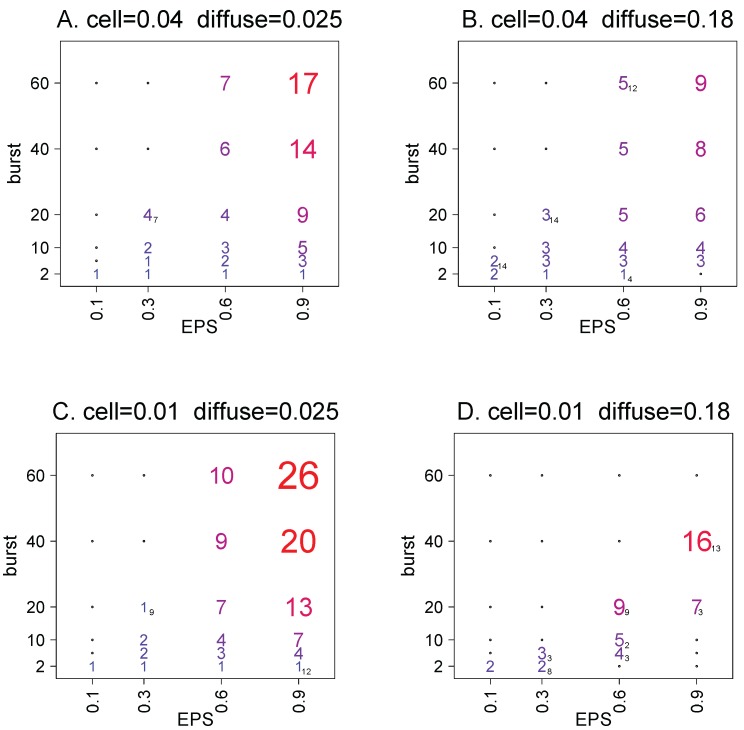
The density of cells maintained in the presence of phage is often increased by spatial structure. Shown in each panel are the $A_{g}$ values, giving the fold increase in cell density over that with mass action. $A_{g}$ values are greatly influenced by EPS levels and burst sizes, exceeding 10 only in the upper right quadrant, with large bursts and high EPS densities, and then only for some values of diffusion and cell reproduction rate. Values within each panel give average $A_{g}$ values from 15 trials each using the same burst and EPS levels, with rate of cell reproduction and phage diffusion rate given at the top of each panel; trials leading to extinction of phage or cells are not included in the averages. EPS was assigned randomly to each patch at the start and remained in the patch for the life of the run; superinfection of infected cells was not allowed ($k_{I}=0$), nor was debris attachment ($k_{D}=0$). Each trial ran 10,000 time steps, and *A* was averaged over the last 3000 steps; values are rounded to the nearest integer (values rounded to 1 were often less than 1). A black subscript denotes the number of trials with bacterial and/or phage extinction; a dot indicates that all 15 trials led to extinction. The ‘cell=’ value given above each panel is the probability that an uninfected cell reproduced at each time step; the ‘diffuse=’ value is the fraction of phage that left the patch in each time step. In all trials, the adsorption probability to uninfected cells was $k_{C}=0.25$, and that to EPS was $k_{E}=0.35$.

Any effect of spatial structure on cell density, even relative density, is likely to depend on details of phage and cell biology. To look for generalities that transcend specifics, simulations were studied for each of a variety of EPS levels, burst sizes, diffusion rates, and cell growth rates (Figure 1). There are in fact general trends, especially that spatial structure often leads to higher cell densities than mass action, but only under some conditions, especially large phage burst sizes.

In each trial, our measure of relative cell density, $A_{g}$, as well as αb and cell density were averaged over the last 3000 steps of runs lasting 10,000 steps, so that the system should have been approaching its equilibrium behavior and any fluctuations would be averaged out. These trials disallowed superinfection of infected cells and attachment to debris: as explained above, this allows calculation of the cell density expected under mass action ($\hat{C}$). An otherwise equivalent set of trials was run allowing superinfection; the αb values were largely unaffected by superinfection, nearly always differing in the first or second decimal place.

Figure 1 shows averages of $A_{g}$ from 15 trials with different random number seeds and three initial conditions (the averages shown exclude extinctions). These $A_{g}$ averages sometimes exceeded 1 by more than an order of magnitude, but were also less than 1 for some parameter combinations (as Figure 1 rounds to the nearest integer, values between 0.5 and 1 are not evident). Not all parameter combinations led to sustained coexistence of bacteria and phage, and parameter combinations leading to extinctions for all 15 trials are omitted from the figure. The largest effects on $A_{g}$ were from changes in EPS and burst size, but changes in the other parameters also had detectable effects. Some of the effects are easily appreciated; for example, it is expected and observed that higher diffusion rates will shift $A_{g}$ toward 1, as the system gets closer to mass action—if cells and phage in fact coexist.

As expected from previous work [16,17], these systems did not always go to a static equilibrium. The trials recorded distributions of αb and $A_{g}$ values for the last 3000 time steps; the distributions were narrow for many parameter combinations but were large for some others. There was no suggestion that high αb or $A_{g}$ was due to large (or small) oscillations, a point that will become reinforced when considering spatially clustered EPS (below). For example, for trials in the upper right corner of Figure 1C (the highest $A_{g}$ averages observed), 80% of the αb values from the run were usually contained in a range spanning 1.0 around the average. In general, there was wider variance in αb with larger bursts and small EPS values. Within the same figure panel (the same cell reproduction and diffusion rates), there was wider variance the closer the burst size and EPS values approached the extinction zone in the upper left quadrant, although trials with burst sizes of 2 and 6 typically did not show a wide variance.

All trials in Figure 1 used the same attachment probabilities, $k_{C}$ and $k_{E}$. To see if the patterns generalize, additional trials considered different combinations of attachment rates for three burst sizes and two EPS values (Table 1); diffusion and cell reproduction rates were those of Figure 1C, and superinfection was again precluded. There is overlap in $A_{g}$ values between burst sizes of 2 and 10 and between 10 and 60. Within an EPS level, the smaller $A_{g}$ value is associated with the smaller burst (with one exception). However, there does not appear to be any single variable strongly determining $A_{g}$ value across all variables. It is also clear that both large and small $A_{g}$ values are not limited to the attachment rates used in Figure 1.

To address the possibility that the observed $A_{g}$ values are bounded artificially by the model, Table 1 includes the upper-bound $A_{g}$ value for each set of parameters, $A_{ub,g}$. In some cases, the observed $A_{g}$ is indeed near its upper bound, raising the possibility that the observed value would be higher with a model structure allowing a higher limit. However, not all high $A_{g}$ values appear to be constrained in this way. This argument will be addressed further when the model is modified to cluster EPS.

The table includes a parallel set of trials and corresponding $A_{g}$ values for mass action in the grid model; the ratio of $A_{g}$ for spatial structure over that for mass action is explicitly the ratio of average cell densities maintained under the two conditions, an empirical comparison that bypasses any use of $\hat{C}$. The major difference between mass action and spatial structure is extinction of the former. For the mass action trials that avoided extinction, none of the spatially structured counterparts had $A_{g}$ averages as high as 2.0.

#### 6.3.2. Understanding the Puzzle of Why Larger Phage Burst Sizes Lead to Higher Cell Densities

The results show clearly that some sets of parameter values lead to large elevations of cell density. The next step is to understand how this elevation happens. In particular, some patterns seem to defy intuition, such as why our relative cell density measures ($A_{g}$ and αb) increase with *b* when holding other parameters constant. It is clear that increasing burst size will affect whether cells and phage are both maintained indefinitely, but the fact that αb changes with *b* indicates that some properties of the infection do not scale proportionally with burst. (αb is more easily addressed in this respect than is $A_{g}$.) Changing EPS abundance is also expected to affect coexistence, but the reason for its affect on αb is not clear. Understanding this absence of proportionality is potentially critical to understanding the effect of spatial structure on cell density, and is addressed next.

To understand how spatial structure enables $A_{g}$ (and thus αb) to exceed 1 and why $A_{g}$ varies with *b*, additional statistics were calculated for the parameter combinations used in Figure 1C (Table 2). The statistics included (i) losses of phage to EPS, (ii) the spatial association of cells and phage with EPS (probability that an uninfected cell or free phage was found in a patch with EPS), and (iii) the proportion of infections that happened in patches with EPS. As true of Figure 1, (i) all statistics were averaged over the last 3000 time steps of 10,000 step runs, and (ii) all statistics were averaged over all runs that led to coexistence.

One striking observation is that, holding all other parameters constant, increases in burst size led to directly corresponding increases in phage lost to EPS, while the losses to uninfected cells were only slightly affected. Thus, as burst size increased, the fraction of phage lost to EPS increased disproportionately. Proportionality is expected unless the association of phage or cells with EPS is changing.

A second observation is that uninfected cells are somewhat associated with EPS (the association is often only modestly greater than the fraction of patches with EPS), whereas free phage are strongly associated with an absence of EPS. These latter observations suggest that spatial structure favors the retention of cells and phage into separate refuges where they are differentially protected from loss.

There are also apparent trends that, as burst size increases, (i) an increasing proportion of all infections happen on patches with EPS, and (ii) phage are increasingly associated with EPS. As burst size increases, the phage appears to be spreading to less protected areas and incurring greater loss.

#### 6.3.3. Reasons for Higher Cell Densities Become Clearer When EPS Is Clumped: Cells Have More Protection from Spatial Structure

The patterns seen in Table 1 and Table 2 are somewhat noisy. Those trials assigned EPS randomly to patches across the grid. Although random assignment may be realistic, it may also complicate understanding. Random assignment gives rise to varied and inconsistent boundaries between EPS-containing and EPS-free regions, possibly complicating inferences about associations of phage and cells with EPS. A clustering of EPS into a single area can overcome those difficulties by ensuring that all trials have the same boundaries around EPS. Trials were conducted so that EPS was laid down contiguously within the grid (adjacent rows were filled until the total EPS allotment was reached). This design resulted in a band of EPS on the grid. One straightforward effect of deterministic clustering is that the size of the boundary between EPS and EPS-free zones is now unaffected by the overall level of EPS. Table 3 provides values from a set of runs corresponding to those in Table 2.

Patterns are clearer than with random EPS assignment and support intuition about the effect of spatial structure in enabling high cell densities over those with mass action:

1. $A_{g}$ (αb) is now moderately constant across different EPS levels within the same burst size. The constancy is stronger at smaller burst sizes. This suggests that the width of the EPS zone itself is unimportant to the properties being measured until bursts get large.
2. The span of $A_{g}$ (αb) values across the table is higher than with random EPS, not profoundly so, and some $A_{g}$ (αb) are consistently less than 1, even when $A_{ub,g}$ cannot have imposed the low value. Spatial structure does not invariably increase cell density over mass action.
3. Phage and cells coexist over a wider range of parameter values with clustered EPS than with random EPS. There were no extinctions, in contrast to the many extinctions when EPS was placed randomly.
4. The association of cells with EPS and phage avoidance of EPS is more extreme than with random placement of EPS.
5. There is now a consistent trend that increasing burst size increases the fraction of infections occurring in patches with EPS.
6. Within a burst size, the value $A_{g}$ is far more stable than is the $A_{ub,g}$, suggesting that the observed $A_{g}$ is not often constrained by the upper bound.

**Figure 2:**
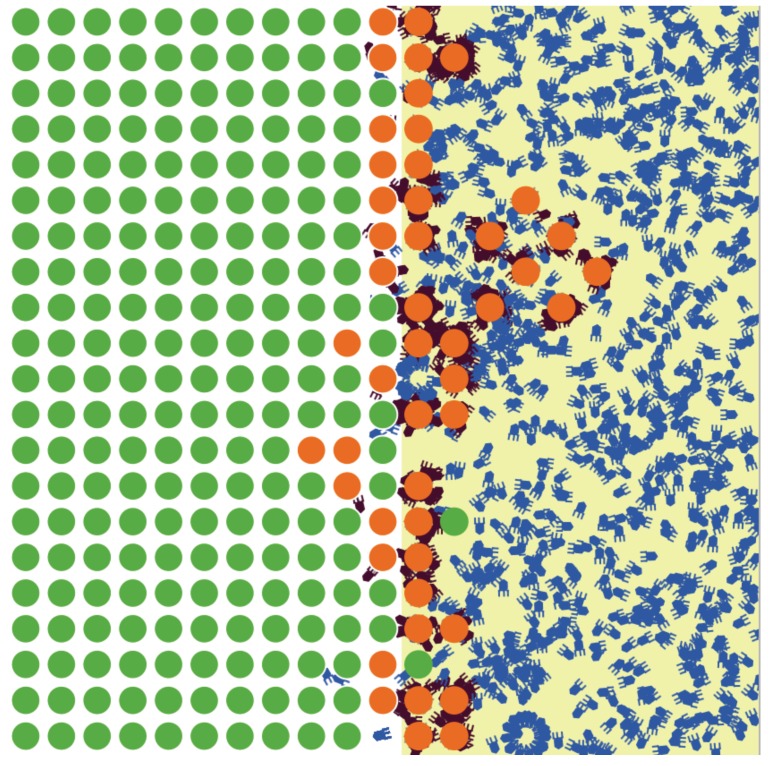
Illustration of self-organization of phages and cells with clumped EPS. White background indicates EPS, yellow is absence of EPS. A green (orange) circle is an uninfected (infected) cell. A blue or black legged icon is a phage (blue is free, black is attached to a cell). Phages are mostly confined to the EPS-free zone and the first row of EPS. Figure was generated from a NetLogo trial with a grid size of 21 × 21, a burst of 20, diffusion step size of 0.45 and attachment probabilities as in Figure 1C. There were 32 phage (partially obscured by cells) in the first three rows of EPS; αb for the entire grid was 7.26.

An intuitive interpretation of these results is that free phage and uninfected cells tend to occupy different patches (phages live in EPS-free patches, cells live in patches with EPS: Figure 2). At low burst sizes, phage are lost to EPS at a high enough rate relative to burst that they virtually only persist in patches without EPS, and they amplify when cells migrate into those patches. This pattern can be argued from the fraction of infections that occur in EPS-free patches. As burst size increases, phages increasingly diffuse into zones with EPS, where they encounter otherwise protected cells. However, these successful infections also result in high rates of phage lost to EPS.

Burst sizes measured from infected cells grown in rich media are often much larger than those evaluated here [1]. However, it should first be appreciated that our simulations of spatial structure are two-dimensional, and a smaller burst size will operate in two dimensions than in three. Since our 2D model characterizes the horizontal spread of phage, it is appropriate to think of only a fraction of the full 3D burst contributing to horizontal spread. Since the volume of a thin slice (say of thickness equal to a tenth of the radius) that intersects the center of a sphere of radius *r* is less than 10% of the volume of the sphere, a full 3D burst *B* should correspond to an analogous 2D burst of size b<B/10. For example, a burst of 60 in two dimensions corresponds to a burst of over 600 in three dimensions.

Nonetheless, trials with burst sizes of 100 and 300 were evaluated for the same EPS levels and adsorption rates as in Table 3. Analyses of these large bursts were reserved for the clumped EPS model because of the repeatability of outcomes provided by this model. The largest $A_{g}$ values were observed for the EPS levels of 0.9: 48 for a burst of 100 and 66 for a burst of 300. Thus, increasing burst sizes several-fold led to only modest increases in $A_{g}$ values. As in Table 3, nearly all phage per burst were lost to EPS. All trials with EPS of 0.1 and a burst of 300 went extinct, revealing that phage can indeed overwhelm cells if the EPS is clustered in small enough patches (no extinctions were observed for the smaller bursts in Table 3). Furthermore, strong oscillations were typical of all trials, again suggesting that, with the larger burst sizes, phages are invading deeper into the EPS-protected refuges. These dynamical effects of large burst sizes on extinction and dynamics would likely disappear with sufficiently large grid sizes (much larger than 10,000 patches) because the zones of EPS protection would be larger and thus require phages to traverse greater distances before reaching the centers of the EPS zones. From the perspective of how spatial structure contributes to an elevated density of cells, larger bursts increase the elevation, but much less than proportionally.

#### 6.3.4. Average Densities under Differential Equation Mass Action Are Also Sometimes Elevated but Not as Much and for a Different Reason

The analysis so far has compared simulated cell densities under spatial structure to densities expected for mass action equilibrium, except for a few trials in Table 1. It is well known that models of mass action dynamics do not obey equilibrium for wide parameter ranges, instead exhibiting either stable oscillations or accelerating oscillations [29]. It is thus possible that average cell densities under mass action will themselves systematically differ from the expected equilibrium. That is, *A*-values for mass action may not equal 1, as has been implied above.

Two efforts were undertaken to calculate *A*-values for mass action: a simulated version of mass action based on adding ‘mixing’ to the spatial grid model, and a version based on an ODE model. The first mass action model merely modified the simulation code of spatial structure so that phage and cells were each assigned grid positions randomly every time step. However, the comparison of *A*-values for spatial structure and mass action is most informative when the *A*-values for spatial structure are well above 1, as those are the only cases in which there appears to be a meaningful effect of spatial structure on cell density. As was shown in Table 1, cell-phage coexistence under mass action was obtained only with parameter values for which the spatial structure $A_{g}$ values were 1–2. (Increasing the grid size 9-fold did not lead to coexistence for any informative combinations either.) The *A*-values under mass action were sometimes higher, but the main result is that mass action extinction was always the outcome for parameter combinations leading to even moderate $A_{g}$ under spatial structure.

The second approach used ordinary differential equation (ODE) models of mass action:

(7) $$\begin{array}{cc} \dot{C} & =rC(1-C/K)-\kappa CP,\\ \dot{P} & =b\kappa C_{L}P_{L}-\kappa CP-\delta P, \end{array}$$

with the “dot” indicating time derivative, parameters in Table 4, and a subscript *L* indicating the value *L* time units in the past. The (1−C/K) term slows bacterial growth as cell density nears *K*, the carrying capacity.

In contrast to the comparison of mass action and spatially structured trials under the grid model, it is not practical to directly compare cell densities between the grid model and an ODE model because of the much higher cell densities enabled by the ODE model. This realization motivates the use of a parallel *A* statistic for the ODE model. To wit, equilibrium cell density under this ODE model is

$$\bar{C}=\frac{\delta }{\kappa (b-1)}$$

and hence it is this quantity that observed cell densities are compared to when defining an ODE-based *A* value:

$$A_{ode}=\frac{C}{\bar{C}}$$

also dimensionless (the subscript ode indicating the ODE model). Its upper limit is

(8) $$A_{ub,ode}=K/\bar{C}.$$

Using ODEs presents the additional challenge establishing a correspondence between attachment probabilities in the grid model to attachment rates in the ODE model. To develop such a correspondence, we used the fact that, over a single unit of time (1 min in ODE corresponds to 1 time step in grid-based model), a phage avoids EPS in the grid-based model with probability $1-k_{E}E$ and in the ODE model with probability $e^{-\delta }$. Thus, $\delta =-\ln(1-k_{E}E)$ is an approximate equivalence. For $k_{E}=0.35$ and E∈0.1,0.3,0.6,0.9, this gives a range for δ of (0.04, 0.38), but the range goes down to 0.005 for the lowest $k_{E}$ and *E* values used in Table 1. A similar basis was used to obtain equivalence between $k_{C}$ and κ; in contrast to the equivalence for EPS, however, cell density is not fixed, so it is necessary to choose a density for the equivalence. Here, that density was the maximum for the system (*K* for the ODE versus 1 in the grid model). For those cell densities and the $k_{C}$ values used in Table 1, κ was in the range ($5\times 10^{-11}$, $3\times 10^{-10}$). For an ODE model scaled per-minute, these are reasonable values [28], although on the low end for some phages [26]. An exact correspondence between mass action and spatial models is not required, of course, because we are interested in whether any realistic mass action process can give high *A*-values; the conversions derived above merely suggest that the ODE model equivalence lies in established regions of parameter space for phages grown in liquid culture.

ODE numerical trials were run for 50,000 time steps using appropriate parameters (Table 5). Many parameter combinations led to expanding oscillations and premature termination of the run (effective extinction). Coexistence of cells and phage was obtained for many runs as well, typically with stable oscillations. For those, average $A_{ode}$ values ranged from slightly above 1 to 10. The average exceeds 1.0 because of the asymmetry in the range of values: $A_{ode}$ periodically goes up to the limit ($A_{ub,ode}$) but can go no lower than 0 (reflecting an asymmetry in the range of bacterial densities).

The highest $A_{ode}$ averages were associated with the highest oscillations in cell densities (up to 18 orders of magnitude for the trial with an average $A_{ode}$ of 10). No attempt was made to evaluate parameter space comprehensively, as our goal was merely to discover whether sustained oscillations resulted in a deviation of $A_{ode}$ from 1.0. In the absence of oscillations, $A_{ode}$ was 1.0, as expected (one example shown).

Summary of ODE model versus spatial grid model. For the differential equation model, an average $A_{ode}$ above 1 is due entirely to sustained oscillations, whereas for the spatial grid model, an elevated $A_{g}$ is not from oscillations but is intrinsic to the dynamics. Thus, the mechanism of high *A*-values are completely different for spatial structure and mass action; in the former, they are intrinsic to the environment and are approximately constant. In the latter, they arise because of oscillations in cell density and the asymmetry of limits on *A*.

#### 6.3.5. Debris: Adding Greater Reality Does Not Change the Trends

The preceding results from the grid model exclude all mechanisms of phage death except irreversible attachment to EPS, and EPS locations and levels were fixed. Other cell-based mechanisms of phage death are plausible and likely temporary. To consider whether our results continue to hold when other phage death mechanisms are present, we expanded the spatial grid model in include cellular debris—remnants of lysed cells that cause phage to bind irreversibly or inject their genomes non-productively. In mass action (liquid culture), this effect appears to be negligible empirically, as phage concentrations are often stable over months ([1], and personal observations). It is unknown whether debris may constitute a greater element of phage death in biofilms and other structured habitats, but we entertain it as an example of a possibly general phenomenon of local phage death resulting from the lysis of cells. Furthermore, the killing effect of debris may be short-lived, thus difficult to detect empirically, but such effects can be studied in the models.

Following [16,31,32], debris was introduced as infected cells that persisted after death (after lysing). In our trials, they were assigned a fixed lifespan, during which they could act as a phage sink in the same capacity as an infected (but unlysed) cell; α is correspondingly recalculated to include this new loss term, and, because of its inclusion, we can no longer use (3) to calculate an expected cell density under mass action. Our presentation is thus of αb instead of *A*, but αb is a suitable proxy. Additionally, superinfection of infected cells was allowed in these trials. The main effect of this debris model is that the dead cell is present after burst and thus is an additional source of death in the patch when phage densities are highest. Even limiting debris longevity to a mere two time steps had a huge effect on shifting the source of phage loss from EPS to debris but had only a modest effect on αb (as well as on coexistence) (Table 6, columns were added to indicate phage lost to debris and infected cells). Coexistence of phage and cells was typically not observed when debris was present and EPS was absent (not shown), but this outcome is necessarily sensitive to debris longevity (our trials assumed a moderately short life for debris).

## 7. Discussion

Phage and their hosts exist in a predator–prey relationship, the dynamics of which have been modeled for over half a century. These models have assumed population structures of well-mixed environments (mass action), both for mathematical convenience and because laboratory studies of phage have used conditions that represent mass action—flasks in shakers and chemostats. However, it is increasingly evident that bacteria grown in biofilms and other spatial contexts are able to persist at much higher densities than apparent from the models, and it is not clear why. This study used a computational approach to investigate the simple question of whether and how spatially structured cell and phage growth might allow higher equilibrium cell densities than with the well mixed conditions of mass action. This question is motivated by empirical observations suggesting that genetically sensitive cells are often profoundly more protected from phage when grown with structure (e.g., biofilms or aggregates) than when grown in liquid. By uncovering the mechanisms behind these high densities, it may be possible to improve the prospects for phage therapy.

Our main findings are:

1. Spatial structure sometimes, but not always, led to cell densities above those maintained at equilibrium under mass action. However, average cell densities under mass action were also often greater than expected at equilibrium. Any effect of spatial structure in elevating cell densities thus appears to be less than an order of magnitude.
2. The mechanisms of ‘elevated’ cell densities are different between spatial structure and mass action. The effect of spatial structure appears to stem from phage and cells dynamically sorting to occupy different patches in the environment, with cells in patches that otherwise kill phage, and phage occupying patches that did not kill them but were largely free of cells. The elevation under mass action arises from sustained oscillations, due to a large dynamic range for A>1 but *A* being bounded to lie above 0.
3. Under spatial structure, increasing burst size was usually observed to increase the relative cell density—to increase the effect of spatial structure in raising cell density—holding other parameters constant. However, a high abundance of environmental protection (EPS) contributed to relative cell density; phage diffusion rates, cell reproduction rates and attachment rates also had influences.
4. The burst size effect was shown to result from a curious effect of the spatial segregation between phages and cells. At higher burst sizes, phages increasingly invaded refuges occupied by cells and suffered proportionally greater losses. Thus, the per capita phage loss to EPS was higher with higher burst sizes, thus accounting for their poorer efficacy in suppressing cell density.

### 7.1. Back to Nature: Do Our Spatial Models Explain What We Observe?

Our efforts were primarily to look for mechanisms that might promote high cell densities as observed in nature. Having found possible mechanisms, the question then turns whether those mechanisms do indeed operate in nature. The latter question is empirical and is a far greater challenge than merely identifying possible mechanisms. Understanding of the empirical side of phage-bacterial dynamics with spatial structure is rudimentary, and our discussion of it is correspondingly speculative. It is premature to suggest that the mechanisms promoting high cell density in our models are empirically important, but they at least suggest directions of inquiry. Indeed, a recent study accounts for bacterial colony survival amid phage attack merely by considering the rate of colony growth versus the rate of phage penetration; when the colony reaches a certain size before phage encounter, it grows faster than the rate at which phage can penetrate—due in no small part to the large number of phages infecting the same cell in the close confines of the bacterial colony [33]. In their model, therefore, cells persist in spatial structure because phages are slow to invade the structure and because many different phage infect the same cell—an effect we intentionally excluded in most of our trials.

The largest effect of spatial structure on cell density observed in our trials is well short of the apparent effects of spatial structure observed in some empirical systems. Furthermore, mass action models were also observed to maintain average cell densities above the expected equilibrium, albeit that this elevated average arises from oscillations. In some natural systems, cells are maintained at densities several orders of magnitude above those in liquid systems. It could be that the cell density increase under spatial structure observed in a numerical trial is artificially bounded by the construct of the model, hence that a more realistic model would exhibit a far higher equilibrium cell density. While a larger grid size or allowing multiple cells per site could increase the dynamic range of *A*, we speculate that a fully 3D system would better capture the large cell densities seen in biofilms that are subjected to phage attack. Imagining our clustered EPS zone as a 2D slice of a biofilm, a 3D version would have a two-dimensional (surface) interface between the protected and unprotected regions. This surface would be more permeable to phage incursions, but the potential gain in cell density in the EPS zone when going from a 2D to a 3D model could vastly increase the dynamic range of *A*.

An alternative interpretation is that empirically high cell densities arise with spatial structure mostly from mechanisms other than those considered here. At one extreme, cells grown with structure may be resistant to infection. This resistance need not even be genetic [23,24,25,26]. Resistance could stem from changes in gene expression that arise when cells are attached to surfaces. Such gene expression changes could lower phage receptor densities or could lead to the secretion of protective layers.

Alternatively, cell protection with spatial structure could be an automatic consequence of limited diffusion and not even involve changes in gene expression. Thus, if cells normally secrete diffusible substances that can form gradients or protective boundaries, spatial structure would allow those gradients to form and protect cells from all sides, whereas liquid culture would not. In contrast, our models allowed protection purely from phage death: cells could escape phage merely because phage were killed before they could attack cells. That phage death was spatially structured, allowing cells to associate with refuges within that structure. Spatial structure offers many possible mechanisms of cellular escape from phages, and our models point a direction toward more biologically comprehensive processes. Empirical progress in understanding bacterial escape will obviously be useful in directing further modeling efforts.

### 7.2. Our Models in Context

Whereas it is straightforward to measure an average cell density with spatial structure any time cells and phage are maintained, it is difficult to use the same approach to determine the cell density that would obtain under the same conditions if phage and cells were fully mixed: the dynamic ranges of cell and phage densities are limited in the simulations, and the oscillations that typically accompany mass action dynamics lead to extinctions in finite populations, even when the average densities are well above the extinction threshold. We thus developed a metric for calculating the equilibrium cell density expected under mass action that could be compared to the cell densities observed in many of the simulations.

For cells to persist amid phages, the cells must either be fully protected from infection (i.e., some form of resistance, genetic or otherwise), or phages must die often enough to keep from overwhelming the cells. We explored the latter process here. Many of our observations as regards dynamics with spatial structure are similar to those of [17], but we took the analysis one step farther by making a comparison of the effect of spatial structure versus mass action on cell density. Another difference is that we did not impose an intrinsic phage death rate, instead allowing phages to die either from sticking to spatially static substances that could in principle be produced by cells (exopolysaccharide, or EPS), or from infection of ‘debris,’ represented here as short-lived parts of dead cells that persist after lysis (inspired by [31,32]). EPS, which is fixed spatially in our model, and thereby allows cells and phages to differentially organize around them, is similar to the fixed refuge model in [17], the main difference being that we have a specific mechanism for inhibiting phage growth. In our model, superinfection results in phage loss; results in our Figure 1 and Table 1, Table 2 and Table 3 specifically precluded superinfection, but parallel trials that allowed superinfection yielded similar outcomes. In [16], superinfection is beneficial to phage since it is assumed to inhibit lysis with a resultant increase in burst size; in [17], there is no superinfection.

Our chief interest in this study was to evaluate the effect of spatial structure on long-term or equilibrium cell density, comparing it to the density expected under mass action. For the purpose of evaluating the effect of spatial structure on phage-bacterial coexistence, Heilman et al. [17] provided a direct comparison of coexistence under the the two conditions. However, oscillations in cell and phage densities under mass action often led to extinction in the grid-based simulations of mass action we attempted, except in cases for which spatial structure appeared to have a small or no elevating effect on cell density. To evaluate the effect of mass action on cell density for cases of interest, we used an ordinary differential equation model, with parameters chosen to correspond to those of the spatial structure model.

To evaluate the effect of spatial structure on cell density, compared to mass action, we used the well-known principle that, in populations at reproductive equilibrium, each individual merely replaces itself, on average—each offspring has one successful offspring during its lifetime (in asexual populations). For a phage with burst size *b*, this means that for each infection of a cell that survives to burst, only one of those *b* progeny will itself establish a surviving infection. We defined α as the ratio of successful infections divided by all sources of free phage loss, hence this equilibrium condition is αb=1. Under some conditions easily implemented in numerical trials, this equilibrium condition can be used to calculate an equilibrium cell density for mass action. The dimensionless statistic *A* was then used as the ratio of observed cell density over the equilibrium cell density under mass action—the ‘amplification’ effect of spatial structure. This statistic could be derived for the grid model (with or without spatial structure) and for the ODE model of mass action, allowing easy comparisons of the effect of different structures.

Across different parameter combinations in the model of spatial structure, grid-based $A_{g}$ values ranged from slightly less than 1 to nearly 30. Thus, spatial structure sometimes conspired to reduce cell density below that maintained with mass action, but also commonly led to an elevation of cell density—depending on parameter values. However, a similar elevation of average density was also observed under mass action whenever the dynamics exhibited sustained oscillations.

A large effect on *A* was from burst size (*b*). It was not immediately clear why increasing burst size should increase the effect of spatial structure on cell density, so various metrics of phage dynamics were analyzed, and a simple explanation was found. The environmental structure allows cells to reside in protected areas (those with EPS) and phages to exist in death-free areas (those without EPS). This is a type of self organization due to the different causes of death for cells (phage kill them) and phage (EPS kills them). When this organization is established, infections result from cells growing into unprotected areas and/or phage diffusing into zones in which they are rapidly killed but where cells reside. The balance between these two processes shifts as burst size is increased—a larger burst means that phages diffuse further into protected-cell zones, but at a cost that more phage progeny are killed. It is also clear that large burst sizes result in the EPS-free zone being essentially devoid of bacteria; this is reflected in a large fraction of infections being limited to the EPS zone. In contrast, with low burst sizes, cells are growing into unprotected zones, where they are killed by phages and where phages do not die (as in Figure 2). In the case of no superinfection or debris attachment, it is also clear that the denominator in *A* decreases as a function of *b*.

### 7.3. Caveats

One potentially important omission from our models is local variation in cell growth rate (as might be mediated by variation in resource concentration). Bacterial growth is known to be important to phage growth (e.g., [1]), with starved cells reducing burst sizes and increasing times to lysis [34]; a change in susceptibility of cell populations at high density requires non-standard models and leads to alternative stable states of the bacterial system even with mass action [35]. Biofilms are thought to be highly structured for resources and consequent cell growth rates [9,36]. To what extent starvation of cells or delayed spread of phages contributes to high cell densities is not addressed by our model but is certainly a worthy avenue of further analysis. Also excluded are temperate phages, whereby infection can lead to a viable cell carrying the phage genome (a lysogen); dynamics of temperate phages with spatial structure presents a fundamentally different set of challenges [37].

The theory advanced here motivates the empirical search for phage death mechanisms, especially those that operate with spatial structure. We yet know little of how rapidly phage are inactivated by exopolysaccharides, outer membrane vesicles, or other materials produced in situ. Such measurements will be difficult when phages are actively growing on live cells in structured environments, but it should be possible to inactivate cells while leaving the structure intact, then measuring the effect on phages.

## 8. Methods: Simulation Model Basics

Three computer programs were used to model spatial dynamics: a program written in C, a program written in Python, and a program written in NetLogo. Due to its superior runtime and versatility, the C program was used for all results presented; the Python program was written to verify the C program results. The Netlogo program was used early in the study to visualize spatial dynamics and develop intuition about the processes. All three models are broadly similar to those in [16,17].

The C code was also adapted to model mass-action dynamics in a grid model. This version of mass action allows an “apples-to-apples” comparison of mass action and spatial dynamics on the same computational platform—including finite population size and identical parameters. With finite population size, the grid based mass action model is stochastic and thus differs somewhat from an ODE-based mass action model. (The randomness actually disappears in the limit as population size goes to infinity.) Aside from the randomness and heightened probability of extinction due to finite population size in simulations of grid-based mass action, they produce similar behavior to numerical solutions of ODE-based mass action.

*C program for spatial grid model.* The spatial C program was typically run with a 100 × 100 grid of patches with no boundary effects (migration on a torus). Figure 1 and Table 1, Table 2 and Table 3 were generated using this program. All phage, infected cells, dead cells, and EPS were assigned to a patch, and all interactions of phage within a patch occurred with other entities in that patch. A patch could harbor at most one cell (infected or uninfected), but in runs allowing debris (dead cells), a dead cell could occur in a patch with an infected or uninfected cell. Independent phage infection probabilities were assigned to the entities of EPS, cells, infected cells, and dead cells, such that a phage could remain uninfected or infect only one of the other entities. Once infected, cells had a finite lifespan (20 steps).

Within a time step, phage migration from a patch was limited to its eight neighbors, with probabilities according to a truncated symmetric, bivariate normal distribution centered on the patch and with a single variance parameter, as follows. Writing

(9) $$f\left(x,y\right)=\frac{1}{2\pi \sigma^{2}}e^{-\frac{\left(x^{2}+y^{2}\right)}{2\sigma^{2}}},$$

if F(z)=P(Z≤z) denotes the cumulative distribution of the standard (1-dimensional) normal, we have the following probabilities for phage diffusion to the eight patches (of side length 1) in the basic neighborhood:

center patch: $A^{2}$ (no diffusion),
each “orthogonal” neighboring patch: AB,
each diagonal patch: $B^{2}$,
where A=2F(0.5/σ)−1 and B=F(1.5/σ)−F(0.5/σ).
These values were normalized by dividing each by $C=A^{2}+4AB+4B^{2}$ to give the fractions of phage diffusing and remaining in the central patch.

In our trials, most of the probability was to remain on the central patch (no diffusion), so a phage was unlikely to move to a neighboring patch in a single time step. Phage diffusion was calculated deterministically (assigning appropriate fractions of the phage in a patch to that patch and the eight neighboring patches), but the overall net effect of migration on the patch was converted to an integral value by assigning any decimal fraction to 0 or 1 with a random draw in proportion to its magnitude.

Cell reproduction was permitted in every time step, each cell’s reproductive fate chosen randomly according to a fixed probability, and independently of other cells’ fates. Cells could reproduce only if one or more of their eight neighboring patches were unoccupied by a live cell (infected or uninfected), and preference was given that a daughter cell move into an orthogonal (off-diagonal) patch. All runs began with cells distributed randomly to 30% of patches and phage distributed randomly to 30% of patches (a patch getting phage received a burst size of phage).

*C program for mass action grid model.* For mass action, the C program was altered in three ways: (i) after burst and before new infections were allowed, all individuals in the entire population of phage were randomly assigned to patches in the grid; (ii) localized phage diffusion was turned off; and (iii) after cell reproduction, the entire population of infected and uninfected cells was reassigned to new patches, with at most one cell (infected or not) per patch. All other aspects of the mass-action C code are identical to those in the spatial C code, allowing us to assess the effects of spatial structure using the same computational platform. There was no simulation of mass-action dynamics in the case of spatially clumped EPS since only the amount of EPS makes a difference in this case.

*Python program for spatial grid model.* The second spatial simulation, written in Python, assumed a 20 × 20 grid of patches without boundary effects. This simulation served as a prototype for the C simulation, and operates similarly with some exceptions. During each time step in the simulation, following a randomized order, each patch executed cell lysis (if applicable), cell reproduction, infection, and phage diffusion. Then, the simulation repeated the same steps in the next randomly-selected patch until all patches were updated for that time step. Contrast this process with the C simulation, where a single event (e.g., lysis) executed across all patches before the next type of event (e.g., reproduction) executed. In the Python simulation, phage and cells only diffused to orthogonal patches. Allowing for diagonal diffusion did not qualitatively impact the simulation results, as long as both phage and cells followed similar diffusion rules. Early simulations in which cells were allowed to diffuse diagonally (but phage were not) decreased the proportion of infections (I:E) that occurred in EPS under deterministic EPS clustering, and also made I:E sensitive to EPS abundance. Such disparity in diffusion capabilities of phage and cells was determined to be unrealistic, so in the C version of the program, both cells and phage were allowed to diffuse both orthogonally and diagonally. In summary, the differences between the Python and C simulations are minor, and both simulations produced comparable output.

*NetLogo program for spatial grid model.* The third spatial simulation, written in the agent-based platform NetLogo, assumed a 51 × 51 grid of patches without boundary effects. This discrete-time simulation updates all patches simultaneously according to probabilities that are based on the current configuration. It is similar to the C simulation except for the following: (a) individual phage diffuse randomly and independently by taking steps in random directions with a prescribed step size; (b) nutrient-dependent cell growth and lysis, where an initial allocation of nutrient was provided and then replenished periodically by pulsing in fresh nutrient across the grid (though the simulations used here had nutrient pulsing every time step to match the nutrient-independent dynamics of the other two simulations); (c) the offspring of a reproducing cell is placed at one of the eight neighboring patches as long as there is space available. Reproduction is suppressed whenever all these local patches are at their carrying capacity; and (d) an approximation to mean-field dynamics is simulated by using large phage step size and random placement of cell offspring (but no subsequent cellular diffusion). Trends observed with the NetLogo program were similar to those with the other two programs.

The choices of a 20 × 20 grid size for the Python simulation, a 51 × 51 grid size for the NetLogo simulation, and a 100 × 100 grid size for the C simulation were made because of computational constraints but are arbitrary. An increase in grid size moderately increased αb in some conditions and decreased it in others. However, the magnitude of these changes was small, and the larger grid size simulations showed smaller variances in αb than in smaller grid size simulations. For example, in one set of simulations with the C program (EPS =0.9, burst =60, random placement of EPS), αb was 18.19, 17.98, 17.96 at grid sizes of 30 × 30, 100 × 100, and 300 × 300, respectively. Thus, the choice of grid size does not affect the overall trends in αb described here.

Numerical ODE trials were carried out with Mathematica 11.1.0 (Wolfram Research Inc., Champaign, IL, USA) using NDSolve.

## 9. Conclusions

Phages are predators of bacteria. Their predator-prey dynamics have been studied for decades in the ideal conditions of liquid culture, where a reasonable agreement has been obtained between models and observations. More recent studies of phages and bacteria grown on surfaces and other ‘structured’ environments suggest that bacterial densities are often much higher than expected from liquid culture results.

Our study focused on the simple question of how spatial structure alone might allow densities of sensitive cells to be maintained at higher levels than in liquid. Our approach relied on computational models in which bacteria could escape phage only by residing adjacent to environmental phage traps, such as exopolysaccharide or cellular debris that irreversibly binds phage. We found that these types of environments could enable an elevation of cell density in which phage and cells self-organized into different regions of the environment: cells persisted in protected areas, phages persisted in areas that lacked phage-killing agents. However, the magnitude to which cell densities were elevated was always less than 2 orders of magnitude, often less than one order—and less than reported in empirical contexts. Other mechanisms are thus needed to account for bacterial survival amid phage attack in structured environments. 

## Figures and Tables

**Table 1:** Effect of attachment probabilities on cell density in grid models.

				Spatial			Mass Action		
Burst	EPS	$k_{C}$	$k_{E}$	$A_{g}$	ext		$A_{g}$	ext	$A_{\mathrm{ub},g}$
2	0.3	0.05	0.05	1.2	-		1.4	-	3.3
2	0.3	0.05	0.15	0.5	-		1.0	-	1.1
2	0.3	0.05	0.25	0.3	-		-	10	0.7
2	0.3	0.15	0.05	3.1	-		-	10	10.0
2	0.3	0.15	0.15	1.3	-		-	10	3.3
2	0.3	0.15	0.25	0.8	-		1.1	1	2.0
2	0.3	0.25	0.05	5.1	-		-	10	16.7
2	0.3	0.25	0.15	2.2	-		-	10	5.6
2	0.3	0.25	0.25	1.4	-		-	10	3.3
2	0.9	0.05	0.05	-	10		1.0	-	1.1
2	0.9	0.05	0.25	0.2	9		-	10	0.2
2	0.9	0.15	0.05	3.3	8		-	10	3.3
2	0.9	0.15	0.15	1.1	7		1.0	8	1.1
2	0.9	0.15	0.25	0.7	7		-	10	0.7
2	0.9	0.25	0.05	5.5	6		-	10	5.6
2	0.9	0.25	0.15	1.8	7		-	10	1.9
2	0.9	0.25	0.25	1.1	9		-	10	1.1
10	0.3	0.05	0.15	0.8	-		-	10	10.0
10	0.3	0.05	0.25	0.7	-		-	10	6.0
10	0.3	0.15	0.15	1.8	2		-	10	30.0
10	0.3	0.15	0.25	1.3	-		-	10	18.0
10	0.3	0.25	0.15	0.9	4		-	10	50.0
10	0.3	0.25	0.25	2.0	-		-	10	30.0
10	0.9	0.05	0.05	4.4	-		-	10	10.0
10	0.9	0.05	0.15	2.9	-		-	10	3.3
10	0.9	0.05	0.25	1.9	-		1.1	4	2.0
10	0.9	0.15	0.05	9.7	-		-	10	30.0
10	0.9	0.15	0.15	7.7	-		-	10	10.0
10	0.9	0.15	0.25	5.5	-		-	10	6.0
10	0.9	0.25	0.05	14.4	-		-	10	50.0
10	0.9	0.25	0.15	12.2	-		-	10	16.7
10	0.9	0.25	0.25	9.2	-		-	10	10.0
60	0.9	0.05	0.15	8.8	-		-	10	21.9
60	0.9	0.05	0.25	8.2	-		-	10	13.1
60	0.9	0.15	0.15	17.3	-		-	10	65.6
60	0.9	0.15	0.25	18.0	-		-	10	39.3
60	0.9	0.25	0.15	31.4	1		-	10	109.3
60	0.9	0.25	0.25	25.0	-		-	10	65.6

Average amplification of cell density ($A_{g}$) due to spatial structure compared to the amplification under mass action across a range of EPS values, burst sizes, and attachment probabilities ($k_{C}$, $k_{E}$). Columns 5 and 6 are for spatial structure, 7 and 8 for mass action. For each combination, the $A_{g}$ shown in the row is the mean of 10 runs differing in the random seed and spanning 2 different initial concentrations of phage and bacteria (extinctions were excluded from the averages, and superinfection was not allowed). Both EPS values (0.3, 0.9) were tested at each burst size (2, 10, 60) for each possible combination of $k_{C}$ and $k_{E}$ in (0.5, 0.15, 0.25); rows are omitted when all 10 trials resulted in extinction for both mass action and spatial structure (17 cases, including all nine trials with a burst of 60 and EPS value of 0.3); numbers of extinctions are otherwise given when more than 0. $A_{g}$ modestly exceeds 1.0 due to oscillations in density being asymmetric around 1.0. The mass action assumptions were applied in the grid model, so the model parameters are directly comparable except that cells and phage were randomly assigned to locations each generation.

**Table 2:** Spatial grid model outcomes with random placement of EPS, no superinfection or debris.

Burst	EPS	$A_{g}$	$A_{\mathrm{ub},g}$	αb	P→E	C:E	P:E	I:E
2	0.1	0.9	7.1	0.9	1.0	0.2	0.01	0.10
2	0.3	1.0	2.4	1.0	1.0	0.5	0.02	0.19
2	0.6	1.1	1.2	1.0	1.0	0.6	0.03	0.25
2	0.9	0.8	0.8	0.9	1.0	0.9	0.04	0.27
6	0.3	1.9	11.9	1.6	5.0	0.5	0.02	0.34
6	0.6	2.9	6.0	2.2	5.0	0.8	0.06	0.51
6	0.9	3.9	4.0	2.6	5.0	0.9	0.09	0.59
10	0.3	1.9	21.4	1.8	9.0	0.4	0.02	0.37
10	0.6	4.3	10.7	3.2	9.0	0.8	0.07	0.58
10	0.9	6.9	7.1	4.3	9.0	0.9	0.12	0.69
20	0.3	0.5	45.2	0.5	18.6	0.4	0.02	0.39
20	0.6	6.8	22.6	5.2	19.0	0.8	0.08	0.65
20	0.9	13.2	15.1	8.2	19.0	0.9	0.17	0.79
40	0.6	9.5	46.4	7.8	39.0	0.7	0.08	0.68
40	0.9	20.4	31.0	13.7	39.0	1.0	0.24	0.87
60	0.6	10.1	70.2	8.8	59.0	0.7	0.08	0.67
60	0.9	26.1	46.8	18.4	59.0	1.0	0.28	0.90

For these numerical trials, parameter values and initial conditions were as in Figure 1C. For each combination of burst size and EPS, the output values shown in the row are the means of 15 runs differing in the random seed and spanning three different initial concentrations of phage and bacteria. All four EPS values (0.1, 0.3, 0.6, 0.9) were tested at each burst size (2, 6, 10, 20, 40, 60); values are not shown when all 15 trials resulted in extinction. The numbers of extinctions for the data shown are given in Figure 1. Burst is phage burst size. EPS is the fraction of grid sites containing EPS, assigned randomly. $A_{g}$ is the magnitude to which total grid cell density is increased above that expected with mass action. P→E is the average number of phage per burst lost to EPS. C:E is the fraction of uninfected cells found in patches with EPS. P:E is the fraction of free phage found in patches with EPS. I:E is the fraction of infections occurring in patches with EPS.

**Table 3:** Spatial grid model outcomes with deterministically clustered EPS, no superinfection or debris.

Burst	EPS	$A_{g}$	$A_{\mathrm{ub},g}$	αb	P→E	C:E	P:E	I:E
2	0.1	0.7	7.1	0.8	1.0	1.0	0.00	0.274
2	0.3	0.7	2.4	0.8	1.0	1.0	0.00	0.273
2	0.6	0.7	1.2	0.8	1.0	1.0	0.00	0.276
2	0.9	0.7	0.8	0.8	1.0	1.0	0.00	0.272
6	0.1	3.3	35.7	2.4	4.9	1.0	0.00	0.594
6	0.3	3.5	11.9	2.5	4.9	1.0	0.00	0.593
6	0.6	3.5	6.0	2.5	4.9	1.0	0.00	0.594
6	0.9	3.5	4.0	2.5	5.0	1.0	0.01	0.600
10	0.1	5.8	64.3	3.9	8.9	1.0	0.00	0.699
10	0.3	6.2	21.4	4.1	8.9	1.0	0.00	0.698
10	0.6	6.3	10.7	4.1	8.9	1.0	0.00	0.697
10	0.9	6.3	7.1	4.1	9.0	1.0	0.01	0.703
20	0.1	11.7	135.7	7.6	19.0	1.0	0.00	0.805
20	0.3	12.9	45.2	8.1	19.0	1.0	0.00	0.805
20	0.6	13.2	22.6	8.2	19.0	1.0	0.00	0.805
20	0.9	13.4	15.1	8.3	19.0	1.0	0.01	0.804
40	0.1	22.4	278.6	14.6	39.2	1.0	0.00	0.889
40	0.3	26.0	92.9	16.0	39.2	1.0	0.00	0.889
40	0.6	26.9	46.4	16.3	39.2	1.0	0.00	0.890
40	0.9	27.2	31.0	16.4	39.0	1.0	0.02	0.887
60	0.1	30.5	421.4	20.5	59.4	1.0	0.00	0.935
60	0.3	38.0	140.5	23.5	59.3	1.0	0.00	0.943
60	0.6	40.1	70.2	24.3	59.3	1.0	0.01	0.942
60	0.9	40.8	46.8	24.5	59.0	1.0	0.04	0.939

For these numerical trials, parameter values were as in Figure 1C, except that EPS was laid down deterministically in a single cluster. For each combination of EPS and burst size, the output values shown in the row are the means of 15 trials differing in the random seed, spanning three different initial abundances of phage and cells. The range of values as a percent of the mean obtained from the 15 trials never exceeded 11%, except for P:E (the range reaching as high as 110% of the mean, which was invariably tiny). No extinctions occurred. Notation is as in Table 2.

**Table 4:** Model variables and parameters.

Notation	Description	Units
Variables		
*C*	density of uninfected bacteria	/mL
*P*	density of phage	/mL
Parameters		
κ	adsorption rate of phage to cells	mL/min
δ	loss rate of phage to EPS	/min
*b*	burst size of phage	
*L*	lysis time	min
*K*	carrying capacity of environment	/mL

**Table 5:** $A_{ode}$ values for the ODE mass action model.

$A_{\mathrm{ode}}$	$A_{\mathrm{ub},\mathrm{ode}}$	Burst	δ	κ	L	r
3.9–4.1	9.0	10	0.1	$1\times 10^{-10}$	20	0.03
1.7	4.5	10	0.1	$5\times 10^{-11}$	20	0.03
5.8	18.0	10	0.05	$1\times 10^{-10}$	20	0.03
1.1	3.0	10	0.3	$1\times 10^{-10}$	20	0.03
4.2–4.4	9.0	10	0.1	$1\times 10^{-10}$	25	0.03
1.0	1.9	20	0.3	$3\times 10^{-11}$	20	0.03
1.2	3.3	20	0.4	$7\times 10^{-11}$	20	0.03
5.0	9.5	20	0.2	$1\times 10^{-10}$	20	0.03
5.6–5.8	9.8	60	0.3	$5\times 10^{-11}$	25	0.04
3.8	7.4	60	0.4	$5\times 10^{-11}$	20	0.03
10.1–10.2	39.3	60	0.045	$3\times 10^{-11}$	21	0.03

$A_{ode}$ values for a small sample of numerical trials of Equation (7) in which bacteria-phage coexistence was observed for the full 50,000 time units. Parameter combinations leading to extinctions are not shown and often resulted with small changes in a single parameter from a parameter set in which coexistence was otherwise observed. $A_{ode}$ was calculated as the arithmetic mean of cell density divided by δ/(κ(b−1)); averages were calculated every 10,000 time units spanning time 10,000 to 50,000, and when the four values differed, the range is given. Parameters used in the trial are defined in Table 4. Carrying capacity *K* was $10^{9}$ for all trials.

**Table 6:** Random EPS with debris lasting two steps, superinfection allowed.

EPS	Burst	αb	P→C	P→I	P→E	P→D	C:E	P:E	I:E
0.1	6	2.2	1.08	0.71	1.58	2.58	0.17	0.01	0.097
0.3	6	2.4	1.07	0.60	1.83	2.50	0.49	0.02	0.218
0.6	6	2.8	1.11	0.74	1.69	2.46	0.68	0.04	0.257
0.9	6	2.7	1.14	0.83	1.61	2.46	0.90	0.05	0.255
0.1	10	0.3	1.13	1.22	3.51	4.28	0.14	0.00	0.100
0.3	10	3.1	1.13	0.92	3.85	4.10	0.48	0.02	0.272
0.6	10	3.9	1.17	1.04	3.80	3.99	0.76	0.05	0.356
0.9	10	4.4	1.25	1.35	3.44	3.96	0.90	0.07	0.343
0.3	20	3.9	1.24	1.62	9.06	8.09	0.43	0.02	0.297
0.6	20	6.4	1.26	1.42	9.60	7.72	0.77	0.07	0.451
0.9	20	8.7	1.40	1.86	9.16	7.59	0.91	0.11	0.461
0.3	40	4.2	1.47	2.97	19.71	16.12	0.36	0.02	0.272
0.6	40	10.4	1.38	1.88	21.62	15.13	0.76	0.08	0.501
0.9	40	16.4	1.56	2.16	21.62	14.67	0.93	0.17	0.558
0.6	60	13.5	1.47	2.30	33.67	22.55	0.75	0.08	0.510
0.9	60	22.4:	1.63	2.23	34.45	21.69	0.95	0.20	0.616

αb values and other properties of dynamics when debris is included and superinfection of infected cells is allowed. Dead cells persisted for two time steps after cell lysis and acted as a phage sink during this time (adsorption to debris was the same as to live cells, 0.25). Parameter values were otherwise as in Figure 1C. For each combination of EPS and burst size, the output values shown in the row are the means of 15 trials differing in the random seed and using three different initial densities of cells and phage. All four EPS values were tested at each burst size; values are not shown when all 15 trials resulted in extinction. For those rows shown, 10 extinctions occurred for (EPS = 0.9, burst =6), 13 extinctions for (0.1, 10), and two extinctions each for (0.9, 10) and (0.3, 40). Ranges of the 15 values as a per cent of the mean were mostly less than 20% and never exceeded 42%, except that the range of αb values was almost as large as the mean for (0.3, 40); some of those trials experienced large variation in αb values with occasional low numbers of cells. Notation as in Table 2, with P→I indicating the approximate number of phage per burst lost to infected cells and P→D indicating the loss to debris. In contrast to Table 1, Table 2 and Table 3, $A_{g}$ is not provided because the baseline calculation of equilibrium cell density for mass action includes terms that depend on dynamics.

## Abbreviations

The following abbreviations are used in this manuscript:

ODE	ordinary differential equations
EPS	exopolysaccharide
